# PCBs in Older Buildings: Measuring PCB Levels in Caulk and Window Glazing Materials in Older Buildings

**DOI:** 10.3390/environments6020015

**Published:** 2019

**Authors:** Lantis I. Osemwengie, Jade Morgan

**Affiliations:** US Environmental Protection Agency, National Exposure Research Laboratory, Exposure Methods and Measurement Division, P.O. Box 93478, Las Vegas, NV 89193-3478, USA

**Keywords:** PCB, Aroclor®, PCBs in schools, caulk samples, window glazing, extraction methods, pressurized liquid extraction, gas chromatography/electron capture detector (GC/ECD), Accelerated Solvent Extraction (ASE)

## Abstract

A method for the determination of polychlorinated biphenyls (PCBs) in caulk and glazing materials was developed and evaluated by application to a combination of 36 samples of caulk and glazing materials, from four schools in the northeastern area of the United States. Quality control analysis showed a range of 45 to 170% for spike recovery from the various samples and a range of 10.9 to 20.1% difference in precision among replicates. The result for the samples analyzed showed that three of the four schools sampled contained caulking and glazing materials with levels of PCBs >50 μg/g (range 54.6 μg/g to 445,000 μg/g). Across the four schools, 24% of collected caulk and glazing samples contained elevated PCB levels relative to the U.S. Environmental Protection Agency’s (EPA) bulk product waste criterion of 50 μg/g under “The Frank R. Lautenberg Chemical Safety for the 21st Century Act.” The PCBs determined in the samples, exhibited characteristic chromatographic patterns similar to those of Aroclors 1242, 1248, 1254, 1260, 1262, and a 1016/1254 mix.

## 1. Introduction

Polychlorinated biphenyls (PCBs) are known developmental toxicants for mammals [1]. Studies of PCB exposure in humans provide strong evidence for carcinogenicity, neurotoxicity, dermal, and ocular effects; studies in other mammals provide additional evidence for reproductive toxicity, developmental toxicity, hepatotoxicity, and immunotoxicity [2,3]. Before the ban of PCB use in 1979, caulk and glazing materials (mainly polysulfides) were sometimes formulated or mixed with PCB Aroclors for elasticity, making them ideal for sealing windows, door joints, building joints, and seams [4]. Aroclor® types ranging from Aroclor® 1242 through 1262 were commonly used in sealants [5]. These sealants have been demonstrated to be the primary source of PCB contamination in and near buildings, including schools, built before the PCB use ban [6].

In Finland, the amount of PCBs used to prepare sealants has been estimated to vary from 5 to 30% by weight, for a total of 130 to 270 metric tons [7,8]. Studies in Germany, Sweden, and Finland have correlated PCB levels in air, soil, and dust with those in building caulking materials [9–13]. In 2004, using the EPA’s SW-846 Test Method 8082A, Herrick et al., investigated PCB contamination in schools and other buildings in the greater Boston area of the United States [5,14]. One-third of the caulk samples contained PCBs such as Aroclors 1254 and 1260 at levels above 50 μg/g (ranging from 70.5 to 36,200 μg/g), exceeding the EPA criterion of 50 μg/g under “The Frank R. Lautenberg Chemical Safety for the 21st Century Act”, at which a material must be handled and disposed of as PCB bulk product waste [15].

There is little detailed information on the analytical methodology and method performance of the measurement of PCBs in caulk and glazing materials. In 2007, Casey et al. presented a poster demonstrating that accelerated solvent extraction (ASE), a commercially available version of a pressurized liquid extraction system, was a less efficient extraction technique for caulking materials than Soxhlet extraction [16]. Using a sonication extraction technique, Casey et al. detected 337,000 μg/g, 107,000 μg/g and 588 μg/g of PCB Aroclors 1254,1260, and 1248/1254 mixture, respectively, in the analyzed caulking materials. However, no analytical data were provided to allow assessment of the efficacy of the method.

The EPA does not currently have a standard analytical method for PCBs in caulk and glazing materials. Typically, the Soxhlet-based extraction method under EPA SW-846 Test Method 3540C is combined with EPA Test Method 8082A [14]. A search of the literature revealed that the PCB analytical methods for caulk and glazing materials currently used by a few researchers and commercial laboratories lack information needed to evaluate method performance.

The work discussed in this article investigated an enhanced procedure developed from SW-846 Test Method 8082A for the analysis of PCBs in caulk and glazing materials. The method was used to analyze 36 caulk and glazing material samples and 11 quality control (QC) samples from school buildings in the northeastern United States [17]. The samples were collected from interior and exterior doors and windows, interior building seams, around installed fixtures and appliances, and interior and exterior building joints. The sampled caulk and glazing materials came from older school buildings. To analyze these samples, an enhanced method for analyzing PCBs in caulk and window glazing materials (nonconventional sources of PCBs) in older school buildings was developed, evaluated, and generated data for PCBs in these materials [17].

## 2. Experimental

### 2.1. Methods

Recognizing that the flexibility of the caulking and glazing material samples under investigation resulted from their formulation with commercial PCBs or PCB technical mixtures such as Aroclor® 1254 and 1260, we quantified total PCBs by comparing the sample peak recognition pattern with that of the Aroclor® commercial mixture. This approach is considered to be safe because the physical properties (not concentrations) of PCBs were preserved by encapsulation in the caulk and glazing materials, which effectively eliminated severe PCBs degradation by biological agents—i.e., one of several degradation agents.

### 2.2. Materials, Equipment, and Supplies

The analytical method used in this study required a number of PCB Aroclor® standards (Table 1) and organic solvents and materials (Table 2). The equipment and supplies required for analysis of the caulk and glazing material samples are listed in Table 3.

The following methods are detailed below: caulking and glazing material sample collection, sample handling and preparation, sample extraction using pressurized liquid extraction, pre-gel permeation chromatography (GPC) and GPC sample cleanup methods, sulfuric acid wash cleanup, and gas chromatography/electron capture detector (GC/ECD) analysis.

### 2.3. Caulking and Glazing Material Sample Collection

Caulking and glazing material samples were collected from both indoor and outdoor structures from schools (unoccupied during sampling) in the northeastern United States. Caulking and glazing material prevalence, accessibility, and relevance (potential for human exposure) were vital considerations in sample collection. Samples were collected from building areas considered to be the most accessible to school occupants, including classrooms, cafeterias, gymnasiums, libraries, hallways, and stairwells. In most cases, caulking and window glazing materials were widely used throughout the school buildings in some locations, in other cases, the materials were found only in a certain area of a school building. Attempts were made to collect caulking and glazing materials from different floors and multiple types of rooms. Caulk samples were collected from window frames, door frames, and building joints and seams. Window glazing materials, when present, were collected from windows and anywhere they were widely applied in a building.

Duplicate and blank QC samples also were collected. One duplicate sample was collected side-by-side with each test sample. Six field blank samples were created from new silicone and acrylic-latex-silicone caulks and were transported and stored along with the samples. A total of 36 test samples, 5 duplicate samples, and 6 field blanks were collected across the four schools.

Table 4 shows the sampling locations, numbers, and types of samples collected from each of the four schools.

Each caulk and window glazing sample weighed an average of 2.51 g (1.65 standard deviation) and was collected using a fresh set of disposable gloves and a pre-cleaned utility knife, snap-off blade knife, or chisel in 60 mL pre-cleaned, amber glass, wide-mouth vials (I-Chem 340–0060 or equivalent). Zip-lock polyethylene plastic bags were placed under windows containing caulk or glazing materials to prevent any pieces from falling to the ground, thus ensuring that all pieces of caulk or glazing material from the sampled area were collected.

### 2.4. Sample Handling and Preparation

Forty-seven caulk and glazing material samples, including QC samples, were packed in ice and shipped overnight to the EPA Exposure Methods and Measurement Division in Las Vegas, NV, USA, for analysis. The samples were stored in the laboratory refrigerator. Prior to analysis, they were removed from the refrigerator and allowed to gain room temperature.

The various caulk and glazing material samples had different colors and physical properties. The textures of the samples as received varied from flexible to dry and some were flaky. Sample type, collection location, and physical characteristics were noted in the sample record. Based on this information, all samples were separated into five color-based groups: white, yellow, gray, light gray, and dark gray or black. From each sample group, a small slice of sample was cut using a disposable razor blade and placed in nine labeled, 5 mL, wide-mouth vials, to be used for the determination of the most suitable extraction solvent or combination of solvents for PCBs in caulk and glazing materials. Each vial was filled with 2.5 mL of one of the following solvents: toluene; acetone; *n*-hexane; methylene chloride; ethyl acetate; a 1:1 mixture (v/v) of toluene/methylene chloride, acetone/methylene chloride, or *n*-hexane/acetone; and a 3:1 (v/v) toluene/methylene chloride mixture. The 3:1 (v/v) toluene/methylene chloride mixture was prepared due to the slow progress of sorption observed in the 1:1 toluene/methylene chloride mixture.

Each slice from the five color-based sample groups was immersed in one of the five single solvents or the four solvent mixtures for a total of 45 solvent vials. After 24 hours, solvent sorption (indicated by swelling of the caulk or glazing material) was observed, using a magnifying glass, to be greatest for methylene chloride for the flexible materials and for the 1:1 mixture of *n*-hexane/acetone for the dry, flaky materials. In addition, because methylene chloride (mildly polar solvent) is known for its efficient extraction of PCBs, it was used as the most suitable solvent for all flexible caulk and glazing samples [18]. The dry, flaky samples did not show any changes in physical size and were ground and extracted with *n*-hexane/acetone 1:1 (v/v).

### 2.5. Sample Extraction Using Pressurized Liquid Extraction

**Figure 1.**
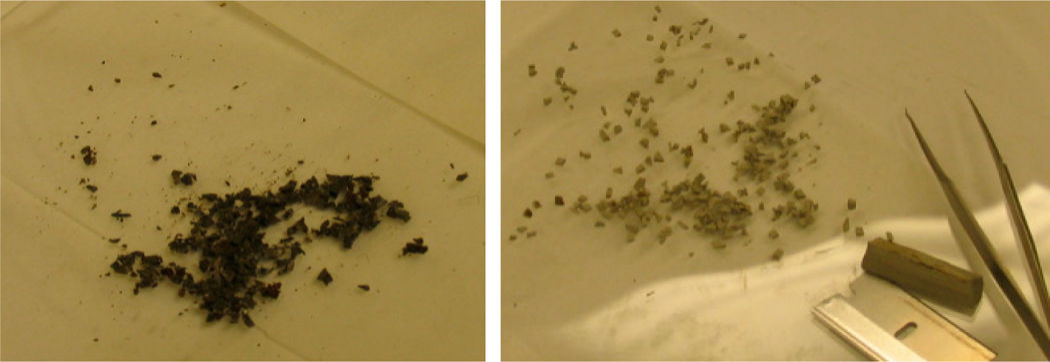
Caulk and glazing materials reduced to smaller particles rising a disposable razor blade or scissors.

Due to the ease of use and large number of samples requiring analysis in a short period of time, an ASE), a commercially available, programmable, high-temperature and high-pressure solvent extraction instrument, was used to perform the extractions. The dry and flaky glazing material samples were ground using a mortar and pestle (both pre-silanized with 5% dimethyldichlorosilane in toluene) to reduce particle size and expose more surface area for ASE using the 1:1 mixture of *n*-hexane/acetone. The flexible caulk samples were sliced into smaller pieces using a disposable razor blade or scissors (Figure 1).

An average weight of 2.5 g of sliced or ground sample material was weighed out using a Mettler AE100 analytical balance for ASE. To determine the extraction efficiency for the caulk and glazing material samples, five samples were extracted using the ASE method described below.

Approximately 2 g of diatomaceous earth hydromatr_i_ix was added to each sliced or ground sample, which was ground or mixed together for even distribution in the sample. This mixture was then transferred to an ASE stainless steel cell using a funnel lined with polytetrafluoroethylene to prevent sample loss. The extraction cell was gently tapped to decrease pore spaces. Using a 500 μL syringe, 100 μL of the surrogate standard tetrachloro-m-xylene at 200 μg/mL was added to each sample [19]. The remaining cell volume was filled with approximately 5 g of alumina for pre-cleanup during extraction.

Each ASE cell then was sealed and placed in the ASE autosampler for extraction in a manner that permitted the extraction solvent to exit the stainless steel cell after passing through 5 g of alumina. The extraction was performed using either a 1:1 mixture (v/v) of *n*-hexane/acetone for the flaky, dried, and ground samples or 100% methylene chloride for the flexible samples under the following ASE conditions: oven temperature 150 °C; pressure 1700 pounds per square inch; static time 5 min; flush volume 60%; nitrogen purge time 60 s; and static cycle set at 2.

### 2.6. Pre-GPC Sample Cleanup Method

Each sample extract was dried by filtering through a glass funnel containing 6 g of pre-baked sodium sulfate and deactivated glass wool directly into a 50 mL TurboVap Zymark concentration tube. Extracts then were filtered through a 0.45 μm polytetrafluoroethylene filter fitted at the end of a 5 mL glass syringe and concentrated to approximately 2 mL using a TurboVap II. Each sample then was solvent-exchanged to methylene chloride and concentrated to 1 mL for GPC. Samples too gelatinous to concentrate to 2 mL for solvent exchange were diluted to 5 or 10 mL, from which 1 mL was taken for solvent exchange and concentrated to 1 mL.

### 2.7. GPC Sample Cleanup Method

A Waters Corporation GPC system equipped with a 515 high performance liquid chromatography pump, a 717 plus autosampler, a 2487 dual-wavelength absorbance detector, and a fraction collector was used for extract purification. The GPC system was fitted with two Envirogel columns in series (19 × 300 mm and 19 × 150 mm), preceded by a Phenogel 10 μm linear/mixed guard column (50 × 7.8 mm). The columns were conditioned with approximately 500 mL of methylene chloride before sample analysis.

**Figure 2.**
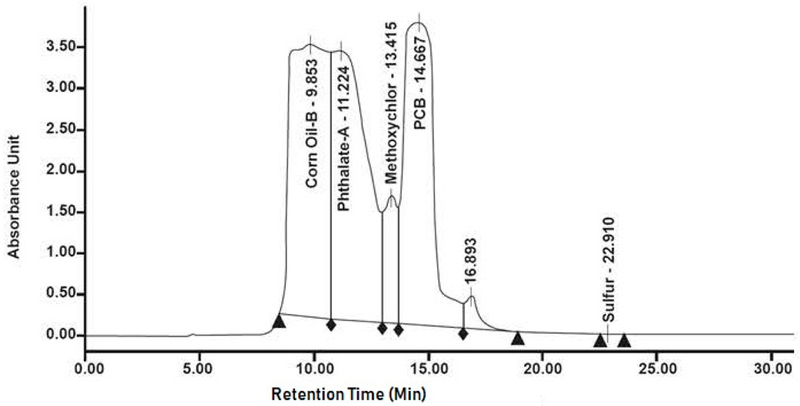
Gel permeation chromatogram of caulk sample extract showing PCB peak elution time at 14.7 min.

To determine collection time windows, the instrument was calibrated using a volume of 50 μL from a solution of 100 μg/mL of Aroolor® 1016tl260 mixture. Metholene chloride was used as the eluting solvent (at specified by the column manufacturer), with a flow rate of 5 mL/min. PCB compounds were found to elute; from the columns between 13 and 20 min in approximately 40 mL of the eluent volume (Figure 2).

As extract fractions eluted from the GPC columns, each fraction was collected in a 50 mL Zymark sample concentration tube located in a fraction collector rack. Using a TurboVap II extract concentration instrument, each sample was blown down to approximately 0.5 mL and quantitatively transferred by glass pipette into a 5 mL, clear glass vial for the sulfuric acid wash.

### 2.8. Sulfuric Acid Wash Cleanup

To each extract, approximately 2 mL of concentrated sulfuric acid was added to decompose the acid labile coextracted compounds and extract any remaining organic compounds into the sulfuric acid. Each vial was placed on a titer plate shaker for 1 min and then placed in a centrifuge for rapid separation at 1000 rpm for 1 min. The clear organic layer was siphoned and pipetted through a homemade column containing 2.5 g of sodium sulfate into a 50 mL Zymark concentration collection tube. This procedure was performed three times consecutively to ensure high analyte recovery and moisture elimination. Extracts were solvent-exchanged to *n*-hexane and concentrated to approximately 4.5 mL each and quantitatively transferred to a 5 mL volumetric flask. Each flask was filled to volume and refrigerated at −4 °C pending GC/ECD analysis.

### 2.9. GC/ECD Analysis

For preliminary analysis, approximately 2 μL of each PCB extract was removed from each 5 mL volumetric flask and injected into the GC/ECD instrument. Extract dilution with *n*-hexane was performed based on the preliminary analytical results. Table 5 shows the dilution factor for each extract.

A 100 μL volume of the internal standard decachlorobiphenyl (1000 μg/mL) was added to 5 mL of each diluted extract before extract injection. The GC/ECD analysis was performed in accordance with EPA Method 608, except different columns and column conditions were used [20]. The GC was equipped with an ECD system (^63^Ni) operating at 320 ° C, with the injector port temperature set at 280 °C. The carrier gas (helium) flow rate was set at 5 mL/min, and the makeup gas (P-5) flow rate was set at 30 mL/min. P-5 represents a mixture of two gases, 5% methane gas and 95% Argon gas. This mixture is used as mobile phase, suitable for the acceleration of post column eluting compounds through the electron capture detector.

**Figure 3.**
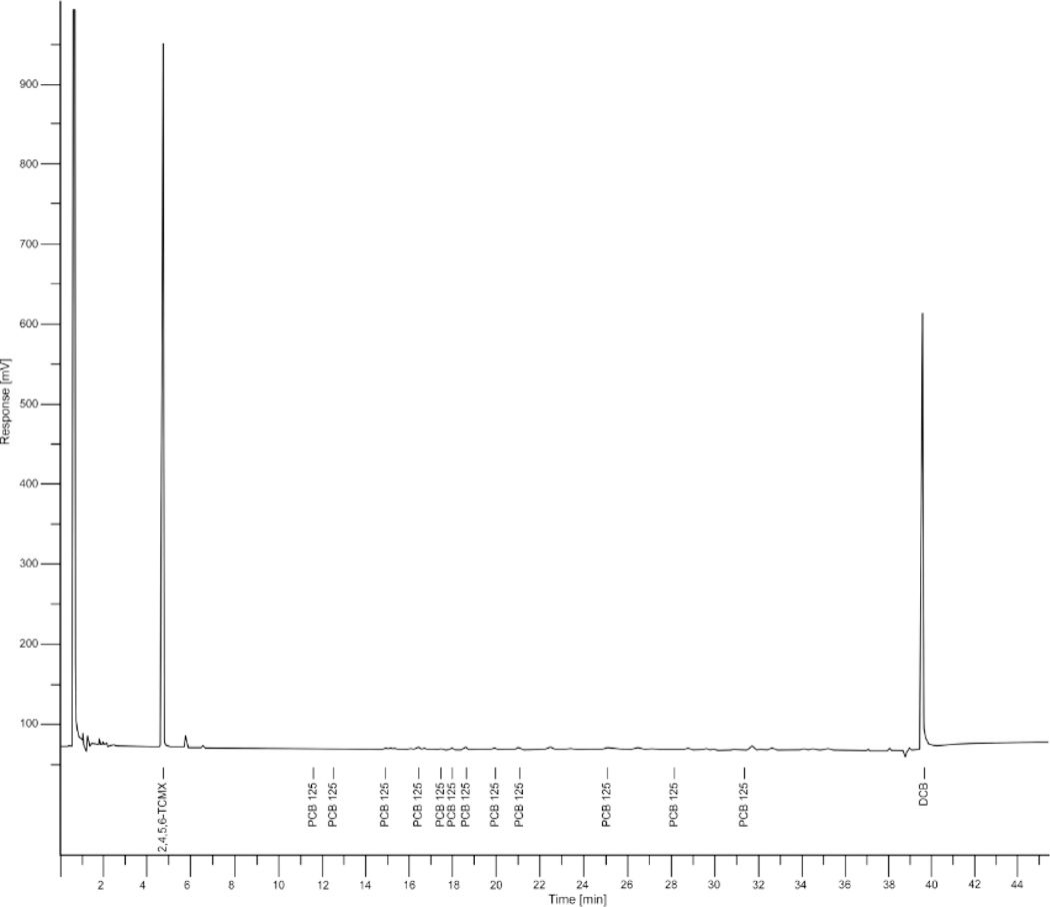
GC-ECD chromatogram of ASE blank extract, showing 2,4,5,6-tetrachloro-m-xylene and decachlorobiphenyl peaks and the elution times.

**Figure 4.**
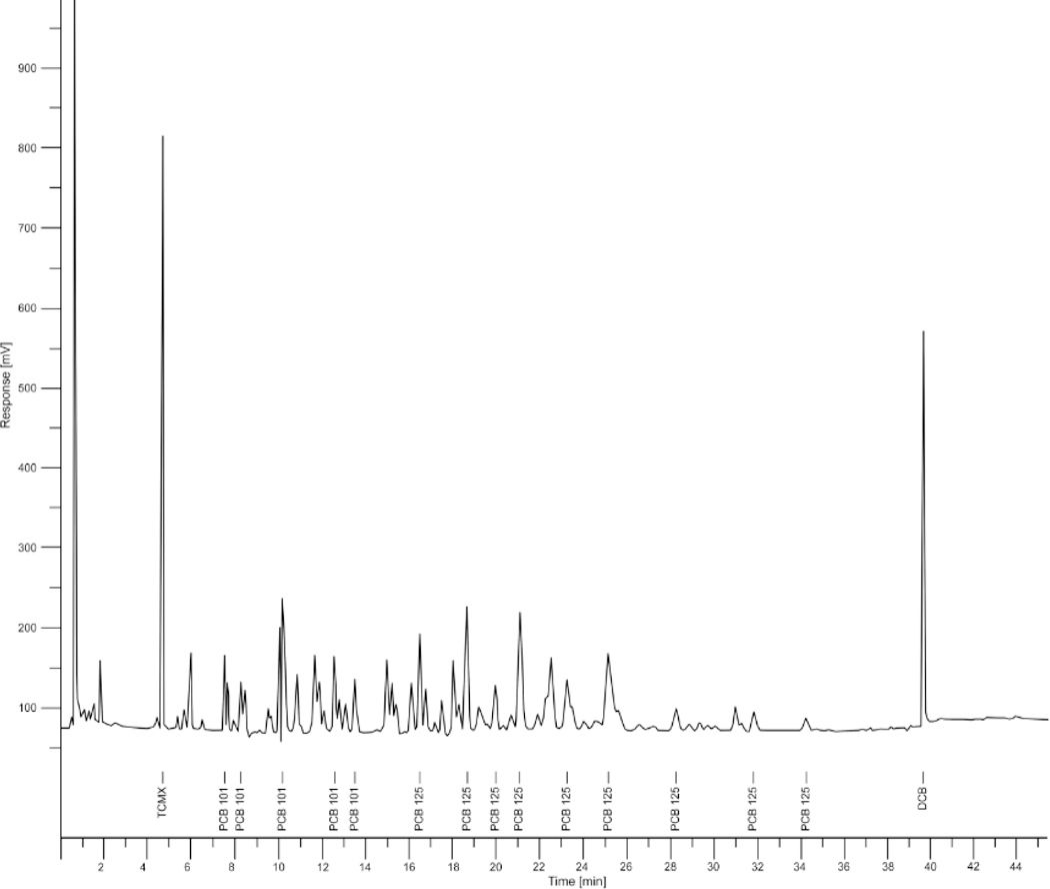
GC-ECD chromatogram of caulk sample extract (WG-10) from school number 1, showing PCB peaks and the elution times.

A 1 μL injection of each final extract was made using the splitless injection mode. The oven temperature was programmed from 150 °C, held for 1 min, ramped from 150 to 200 °C at 3.0 °C/min, held for 10 min, ramped from 200 to 220 °C at 8 °C/min, held for 5 min, ramped from 220 to 300 °C at 15 °C/min, and held for 5 min, for a total run time of 45.5 min. The consistently reproducible individual PCB congener peaks produced from authentic PCB Aroclor® calibration standards were used to quantify the total PCBs. Figures 3 and 4 below, shows the chromatograms obtained from the ASE blank and sample WG-10 from school number 1 respectively.

## 3. Results

As Table 5 shows, PCB characteristic chromatographic patterns similar to those of Aroclors 1242, 1248,1254,1260, and 1262 were detec ted in the caulk: and glazing samples from the schools. Most of the PCB characteristic chromatographic patterns detected in caulk material sample extracts were similar to that of Aroclor® 0254. The method detection limit (MDL) was determined to be 1.2 μg/g from three times the signal-to-noise ratio. Levels exceeding 50 μ/g (54.6 to 445,000 μg/g) were detected in some of the caulk samples, while lower levels (<MDL to 27.5 μg/g) were detected in other samples. It’should be noted that results of the sample analysis were conveyed So the school district, and school representatives opted to immediately remediate the contaminated materials.)

Of the 36 caulk and window glazing samples, 13 (35 %) had PCB levels less than the MDL, 15 (41 %) had PCB levels less than 50 μg/g, an1 9 (24%) had levels greater than 50 μg/g. One window glazing sample contained 7.5 μg/g of Aroclor® 1254, but all the other window glazing sample results were less than the MDL.

The highest PCB concentrations were detected in caulk samples from School 2 as follows:

445,000 μg/ g in one inUeri or caulk sample and a53,000 and 1 31,000 μg/g in two exterior caulk samples. A very high level of PCBs (105,000 μg/g) also was detected in one; exterior building joint caulk sample from School 4. Different types of caulk were found at each school, and the levels of PCBs in these different caulks varied widely between schools.

As shown in Table 6,10 spiked samples, replicated one to seven times each, were used to assess the efficacy of the extraction technique. QC analysis showed a range of 48.8 to 171% for spike recovery from the various samples and a range of 10.9 to 20.1 relative percent difference in precision among replicates.

## 4. Discussion

The high total PCB values for some of the caulk samples obtained using the extraction technique described in this study are comparable to the total PCB range of 124,000 to 327,000 μg/g obtained by Burkhardt et al. using a Soxhlet extraction system [10]. The average percent recovery values for Aroclor® 1254 in the caulk materials coupled with the high PCB concentrations for several caulk material samples in this study indicate how efficient ASE is for extracting high PCB concentrations from caulk and glazing materials. The use of GPC for the initial sample extract cleanup eliminated the need for further cleanup steps using silica and florisil cartridges.

In this study, total PCBs were quantified by comparing the sample peak recognition pattern with that of Aroclor®, a commercial mixture, because the caulking and glazing material samples under investigation are known to have been directly formulated with commercial PCBs or PCB technical mixture. However, to avoid quantification errors, it should be noted that the PCB congener-specific method, using gas chromatography and mass spectrometry, is recommended for biologically degraded PCB samples because of the significant differences in PCB recognition patterns between PCBs in caulk and glazing material samples and PCBs in biologically degraded biological samples. Due to the different stages of degradation and ages of the caulk samples—i.e., over 40 years of age, we believed that the weathered PCB congeners that constitute the Aroclor mixtures may have contributed to the variation in peaks area. This may have resulted in a rather broad range of recovery values.

## 5. Conclusions

The high levels of PCBs found in the caulk samples are consistent with the composition of the original materials used, which contained 5% or higher of PCB Aroclors (assuming a universal production formulation) [9]. This finding combined with the findings of studies in Finland, Germany, Sweden, and the fairly recent review of PCBs in schools’ article by Robert F. Herrick, et al. [21] indicates that the presence of PCBs in caulk in older buildings is an international concern. The sample preparation methods, solvents, ASE technique, and cleanup methods in the enhanced analytical method for caulk and window glazing materials developed from EPA SW-846 Test Method 8082A provided reliable measurement results. The results and method validation may also prove useful in developing nations or low and middle-income countries where PCBs may be found. Further side-by-side evaluations are recommended using the Soxhlet extraction system and other methods.

## Figures and Tables

**Table 1.** PCB Aroclor® Standards.

Standard	Concentration	Manufacturer
Aroclors 1016,1221,1232,1242,1248,1254,1260,1262	1000 ng/μL in hexane	AccuStandard (New Haven, CT, USA)
Aroclor® Mixture 1016/1260	1000 ng/μL in hexane	AccuStandard (New Haven, CT, USA)
Mixtures 1016/1232/1248/1260 and 1221/1242/1254	0.2 μg/mL in methanol	AccuStandard (New Haven, CT, USA)
Reference check standards (Similar to standards above)	1000 ng/μL in hexane	Restek (Bellefonte, PA, USA)
Decachlorobiphenyl (surrogate standard)	1000 μg/mL in toluene	Absolute Standards, Inc. (Hamden, CT, USA)
2,4,5,6-Tetrachloro-*m*-xylene	200 μg/mL in acetone	Restek (Bellefonte, PA, USA)

Abbreviations: Tetrachloro-*m*-xylene. (TCMX).

**Table 2.** Solvents and Materials.

Solvent or Material	Manufacturer

99.5% acetone	Anachemia (Rouses Point, NY, USA)
99.9% ethyl acetate	Spectrum (Gardener, CA, USA)
99.9% *n*-hexane, capillary GC grade	Sigma Aldrich (St. Louis, MO, USA)
100% toluene, HPLC grade	J.T. Baker (Phillipsburg, NJ, USA)
100% methylene chloride, HPLC grade	J.T. Baker (Phillipsburg, NJ, USA)
Concentrated sulfuric acid, lot # 990715	Fisher Scientific (Pittsburgh, PA, USA)
Anhydrous granular sodium sulfate	EMD Chemicals (Gibbstown, NJ, USA)
Glass wool treated with DMDCS	Ohio Valley Specialty Chemical (Marietta, OH, USA)
Diatomaceous earth hydromatrix, part # 198003	Varian Inc. (Palo Alto, CA, USA)

Abbreviations: DMDCS, dimethyldichlorosilane; HPLC, high-performance liquid chromatography; GC, gas chromatography.

**Table 3.** Equipment and Supplies.

Equipment or Supply	Manufacturer
Hettich® Universal 320R centrifuge	Sigma-Aldrich (St. Louis, MO, USA)
Mettler AE100 analytical balance Barnstead Nanopure water system supplying	Mettler Toledo (Columbus, OH, USA)
DI water with resistivity of 17.5 megaohm-centimeter	Barnstead/Thermolyne (Dubuque, IA, USA)
TurboVap II for sample concentration	Caliper Life Sciences (Mountain View, CA, USA)
GPC columns for sample cleanup	Waters Corporation (Milford, MA, USA)
Phenogel 10 μm linear/mixed guard column, 50 × 7.8 mm for protecting the GPC column	Phenomenex (Torrance, CA, USA)
ASE 350 extraction instrument	Dionex (Sunnyvale, CA, USA)
Titer plate shaker, model #4625	Lab Line Instruments (Melrose Park, IL, USA)
Clarus 500 GC	PerkinElmer (Waltham, MA, USA)
Analytical column, 30 m, 0.53 mm inside diameter (ID), Rtx-XLB, catalog #12840	Restek Inc. (Bellefonte, PA, USA)
Confirmation column, 30 m, 0.53 mm ID	Restek Inc. (Bellefonte, PA, USA)
0.5 μm film Rtx-35MS, catalog #14640 Carrier gas, helium, UHP/zero grade, 99.999%	Praxair, Inc. (Danbury, CT, USA)
Makeup gas, P-5, 95% argon and 5% methane	Praxair, Inc. (Danbury, CT, USA)

Abbreviations: DI, deionized; GC, gas chromatograph; GPC, gel permeation chromatography; ID, inside diameter.

**Table 4.** Number and Types of Caulk and Glazing Material Samples Collected from Each School.

	School			
	l	2	3	4

**Sampling Location Description**	**Number of Samples**			
Exterior caulk (EC)	3	2	2	3
Interior caulk (IC)	7	4	4	0
Exterior building joint caulk (EJ)	1	0	0	2
Interior building joint caulk (IJ)	0	1	0	2
Window glazing (WG)	3	0	1	1

**QC Samples**				
Exterior caulk (EC) duplicate	1	1	0	0
Interior caulk (IC) duplicate	0	1	0	0
Window glazing (WG) duplicate	0	0	1	1
Silicone caulk field blank	1	1	1	0
Acrylic latex with silicone field blank	1	1	1	0

**Total samples collected per school**	17	11	10	9

**Table 5.** Sample Number, Dilution Factor, PCB Concentration, and Aroclor® Pattern Detected.

School	Sample No.	Dilution Factor	PCB Concentration (μg/g)	Aroclor® Pattern

1	EC-10	100	720	1242
	EC-10 (duplicate)	200	663	1242
	EC-11	1	8.45	1254
	EC-12	5	7.32	1242
	IC-10	1	<MDL*	
	IC-11	5	14.1	1254
	IC-12	5	1220	1254
	IC-13	100	161	1262
	IC-14	5	16.5	1254
	IC-15	40	90.5	1262
	IC-16	10	13.6	1262
	EJ-10	1	<MDL *	1242
	WG-10	1	7.54	1254
	WG-11	1	<MDL *	
	WG-12	1	<MDL *	

2	EC-10	500	997	1254
	EC-11	30,000	153,000	1254
	EC-11 (duplicate)	10,000	131,000	1254
	IC-10	50,000	445,000	1254
	IC-11	5	17.1	1016/1254 Mix
	IC-12	1	4.41	1254
	IC-12 (duplicate)	1	3.77	1254
	IC-14	5	27	1248
	IJ-10	1	10.3	1254

3	EC-10	1	<MDL *	
	EC-11	5	<MDL *	
	IC-10	1	<MDL *	
	IC-11	1	5.33	1254
	IC-12	1	<MDL *	
	IC-13	5	26.2	1242
	WG-10	1	<MDL *	
	WG-10(duplicate)	1	<MDL *	

4	EC-10	1	1.52	1254
	EC-11	25	54.6	1254/1260 Mix
	EC-12	1	<MDL *	
	EJ-10	1	<MDL *	
	EJ-11	40,000	105,000	1262
	IJ-10	5	27.5	1242
	IJ-11	1	1.73	1254
	WG-10	1	<MDL *	
	WG-11 (duplicate)	1	<MDL *	

1	Field blank	1	<MDL *	
	Field blank	1	<MDL *	

2	Field blank	1	<MDL *	
	Field blank	1	<MDL *	

3	Field blank	1	<MDL *	
	Field blank	1	<MDL *	

Abbreviations: EC, exterior caulk; IC, interior caulk; EJ, exterior building joint caulk; IJ, interior building joint caulk; WG, window glazing.

* MDL = Method detection limit of 1.2 μg/g (three times the signal-to-noise ratio).

**Table 6.** PCB Average Percent Spike Recovery, Relative Standard Deviation, and Relative Percent Difference.

PCB Aroclor®	School	Sample No.	% Spike Recovery	RSD	RPD

1254/1260	4	WG-10	66.2	4.8 (n = 4)	13.3
1016/1254	3	WG-10	48.8	10.8 (n = 7)	17.5
1248	1	EC-10 (dup)	54.9	3.3 (n = 3)	10.9
1254	4	WG-11	66.6	8.3 (n = 2)	17.6
1254	3	WG-10	97	28.0 (n = 4)	20.1
1254	4	EC-12	103	34.0 (n = 3)	11.1
1254	3	EC-10	137	10.7 (n = 2)	11.2
1254	3	EC-11	100	13.0 (n = 2)	18.4
1254	4	IJ-11	137	NA (n = 1)	NA
1254	4	EC-10	171	NA (n = 1)	NA

Abbreviations: NA, not applicable; RPD, relative percent difference; RSD, relative standard deviation.

## References

[1] Kuusisto S; Lindroos O; Rantio T; Priha E; Tuhkanen T PCB contaminated dust on indoor surfaces—Health risks and acceptable surface concentrations in residential and occupational settings. Chemosphere 2007, 67,1194–1201. 17166563 10.1016/j.chemosphere.2006.10.060

[2] Toxicological Profile for Polychlorinated Biphenyls (PCBs); Agency for Toxic Substances and Disease Registry: Atlanta, GA, USA, 2000; 948p. Available online: https://www.atsdr.cdc.gov/toxprofiles/tp.asp?id=142&tid=26 (accessed on 2 March 2017). 36888731

[3] Polychlorinated Biphenyls and Polybrominated Biphenyls; International Agency for Research on Cancer: Lyon, France, 2016; Volume 107, Available online: http://monographs.iarc.fr/ENG/Monographs/vol107/mono107.pdf (accessed on 2 March 2017).

[4] Kohler M; Tremp J; Zennegg M; Seiler C; Minder-Kohler S; Beck M; Lienemann P; Wegmann L; Schmid P Joint sealants: An overlooked diffuse source of polychlorinated biphenyls in buildings. Environ. Sci. Technol. 2005, 39,1967–1973. 15871225 10.1021/es048632z

[5] Herrick RF; McClean MD; Meeker JD; Baxter LK; Weymouth GA An unrecognized source of PCB contamination in schools and other buildings. Environ. Health Perspect. 2004,112, 1051–1053. 15238275 10.1289/ehp.6912 PMC1247375

[6] Rantio T; Riala R; Kontsas H; Back B; Pekari K; Kallio A; Oksa P; Priha E PCB-Ja Lyijypitoisen Saumausmassan Turvallinen Poistaminen Elementtirakennuksista [Safe Remove of PCB- and Lead-Containing Elastic Sealants in Prefabricated Houses]; Finnish Institute of Occupational Health: Tampere, Finland, 2001; 63p. (In Finnish)

[7] Priha E; Hellman S; Sorvari J PCB contamination from polysulphide sealants in residential areas-exposure and risk assessment. Chemosphere 2005, 59, 537–543. 15788176 10.1016/j.chemosphere.2005.01.010

[8] Haukijarvi M; Pentti M Rakennusten Saumausmassat Ja PCB-Yhdisteet [Sealing Compounds of Buildings and PCB]; Tampereen Teknillinen Korkeakoulu: Tampere, Finland, 2000 (In Finnish)

[9] Balfanz E; Fuchs J; Kieper H Sampling and analysis of polychlorinated biphenyls (PCB) in indoor air due to permanently elastic sealants. Chemosphere 1993, 26, 871–880.

[10] Burkhardt U; Bork M; Balfanz E; Leidel J Indoor pollution by polychlorinated biphenyls (PCB) in permanently elastic sealing compounds. Offentl. Gesundheitswes 1990, 52, 567–574. (2149431

[11] Fromme H; Baldauf AM; Klautke O; Piloty M; Bohrer L Polychlorierte biphenyle (PCB) in fugendichtungsmassen von gebauden: Bestandsaufnahme fur Berlin und neue innenraumquellen [Polychlorinated biphenyls (PCB) in permanently elastic sealants in buildings: Stocktaking for Berlin, and for new indoor sources]. Das Gesundheitswesen 1996, 58, 666–672. (In German) 9081511

[12] Pyy V; Lyly O PCB in Mastic Sealants in Prefabricated Houses and in Courtyard Soil; City of Helsinki Environmental Center: Helsinki, Finland, 1998 (In Finnish)

[13] Corner R; Sundahl M; Rosell L; Ek-Olausson B; Tysklind M PCB in indoor air and dust in buildings in Stockholm. In Proceedings of the 9th International Conference on Indoor Air Quality and Climate, Monterey, CA, USA, 30 June-5 July 2002; pp. 141–146.

[14] Method 8082A (SW-846): Polychlorinated Biphenyls (PCBs) by Gas Chromatography; United States Environmental Protection Agency: Washington, DC, USA, 2007; 56p.

[15] Manufacturing, Processing, Distribution in Commerce, and Use of PCBs and PCB Items (US). Fed. Regist. 2011,40, 726–730.

[16] Casey A; Wagner RE; Herold TE; Glenn M The evaluation of extraction and cleanup methods for the determination of PCB aroclors in caulking and sealing material. In Proceedings of the 22nd Annual Conference on Soils, Sediments, and Water, Amherst, MA, USA, 16–19 October 2006.

[17] Thomas K; Xue J; Williams R; Jones P; Whitaker D Polychlorinated Biphenyls (PCBs) in School Buildings: Sources, Environmental Levels, and Exposure; United States Environmental Protection Agency: Washington, DC, USA, 2012; 150p.

[18] Test Methods for Evaluating Solid Waste, Physical/Chemical Methods: SW-846; United States Environmental Protection Agency: Washington, DC, USA, 1998.

[19] Murphy BL; Morrison RD (Eds.) Introduction to Environmental Forensics, 2nd ed.; Academic Press: Cambridge, MA, USA, 2007; 776p.

[20] Methods for Organic Chemical Analysis of Municipal and Industrial Wastewater; United States Environmental Protection Agency: Washington, DC, USA, 1985.

[21] Herrick RF; Stewart JH; Allen JG Review of PCBs in US Schools: A Brief History, Estimate of the Number of Impacted Schools, and an Approach for Evaluating Indoor Air Samples. Environ. Sci. Pollut. Res. 2016,23,1975–1985. [CrossRef] 10.1007/s11356-015-4574-8 PMC4635108 25940477

